# A synthetic molecule targeting STAT3 against human oral squamous cell carcinoma cells

**DOI:** 10.7150/ijms.105200

**Published:** 2025-02-10

**Authors:** Li-Yuan Bai, Eman M. E. Dokla, Po-Chen Chu, Chia-Hsien Feng, Jing-Lan Hu, Liang-Jun Wang, Jing-Ru Weng

**Affiliations:** 1 Division of Hematology and Oncology, Department of Internal Medicine, China Medical University Hospital, Taichung 404, Taiwan.; 2 College of Medicine, China Medical University, Taichung 404, Taiwan.; 3 Pharmaceutical Chemistry Department, Faculty of Pharmacy, Ain Shams University, Cairo 115, Egypt.; 4 Department of Cosmeceutics and Graduate Institute of Cosmeceutics, China Medical University, Taichung 404, Taiwan.; 5 Department of Fragrance and Cosmetic Science, College of Pharmacy, Kaohsiung Medical University, Kaohsiung 807, Taiwan.; 6 Department of Marine Biotechnology and Resources, National Sun Yat-sen University, Kaohsiung 804, Taiwan.; 7 Graduate Institute of Natural Products, Kaohsiung Medical University, Kaohsiung 807, Taiwan.; 8 Graduate Institute of Pharmacognosy, College of Pharmacy, Taipei Medical University, Taipei 110, Taiwan.

**Keywords:** Oral squamous cell carcinoma, apoptosis, migration, MAPK, STAT3

## Abstract

Oral squamous cell carcinoma (OSCC), one of the most common cancers in Taiwan, needs new therapeutic agents and treatments. The aim of this study was to investigate the anti-proliferative activity of {*N*-[3-chloro-4-[5-[3-[[[4-[(cyclopropylcarbonyl)-amino]3-(trifluoromethyl)phenylamino]carbonyl]amino]phenyl]-1,2,4-oxadiazol-3-yl]phenyl]-3-pyridine-carboxamide} (COC), a synthetic molecule, in OSCC cells. COC exhibits potent tumor-suppressive efficacy with IC50 values of 195 nM and 204 nM toward SCC2095 and SCC4 OSCC cells, respectively. Our data revealed that COC caused caspase-dependent apoptosis and downregulated the MAPK signaling pathway. In addition, COC modulated the levels of E-cadherin and β-catenin and inhibited migration. COC also decreased p-STAT3 levels, and the overexpression of STAT3 partially attenuated COC-induced cytotoxicity. Therefore, our findings suggest the use of COC as a new approach to oral cancer treatment.

## Introduction

According to the report of the American Cancer Society (ACS), the estimated number of new cases of oral squamous cell carcinoma (OSCC) is more than 58,000 in 2024, with an increasing trend of 2.3% per year in the USA 1. OSCC is the 2^nd^ leading cause of cancer deaths in South and South-East Asia, and Taiwan is within the top 5 countries owing to the high incidence in the area 2. Tobacco smoking, secondhand smoke, betel nut chewing, heavy alcohol drinking, and human papillomavirus infection are the risk factors for OSCC 3. Currently, there are multiple treatment strategies for OSCC, including surgery, radiotherapy, chemotherapy, and immunotherapy. However, recurrence, severe side effects, trauma, and high hospital charges have negative impacts on the efficacy of therapy and patient compliance 4, 5. Despite the combination of surgery, chemotherapy, and immunotherapy, the 5-year survival rate of patients with OSCC after therapy remains between 25 and 50% 6. Therefore, new drugs or therapeutic strategies are needed.

**Figure 1:**
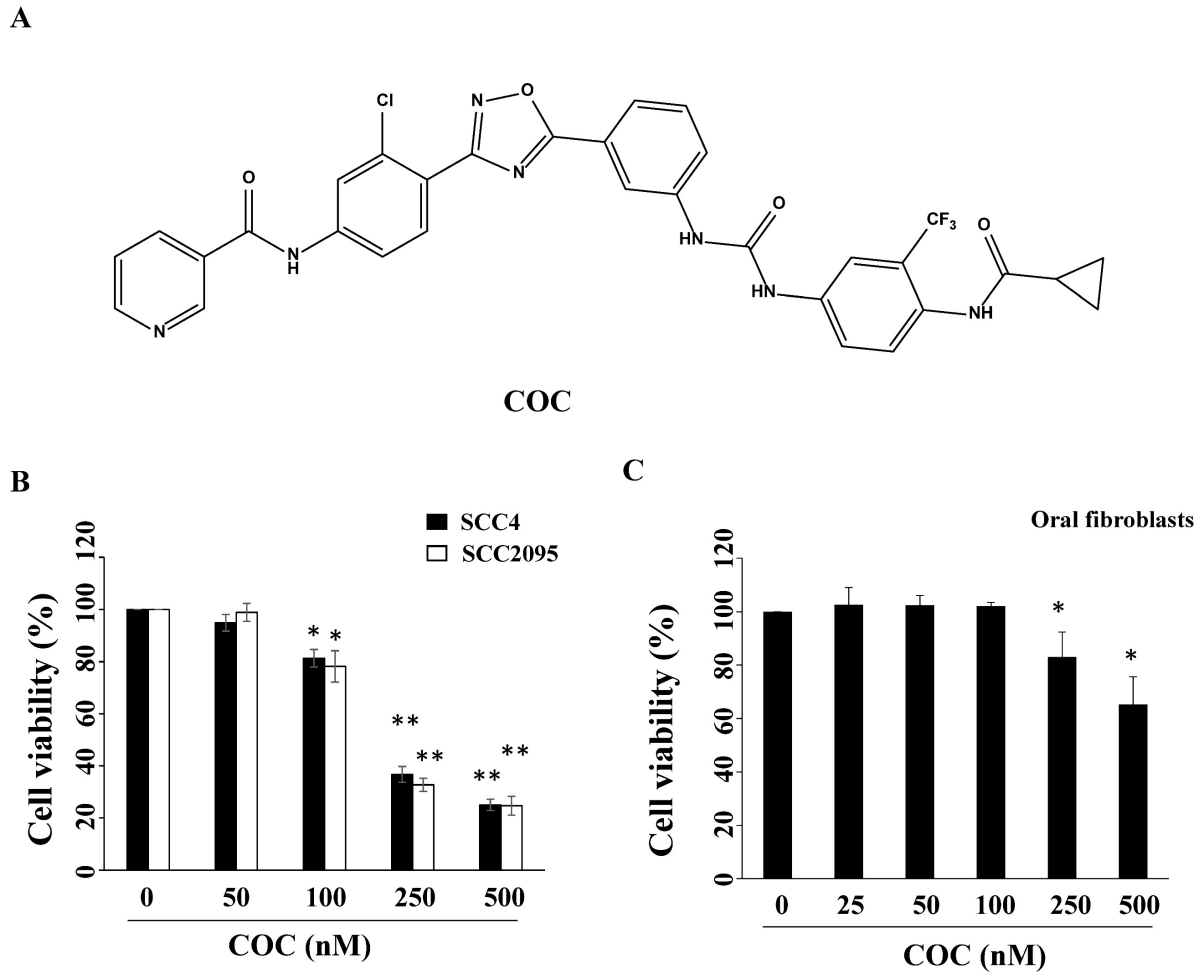
(A) Structure of COC. (B) Cytotoxicity of COC in SCC2095 and SCC4 OSCC cell lines, and (C) oral fibroblasts. Cells were treated with DMSO or COC at different concentrations for 24 h. *P < 0.05, **P < 0.01.

Epidermal growth factor receptor (EGFR) is a member of the human epidermal growth factor receptor tyrosine kinase (RTK) family, which regulates proliferation, cell cycle, migration, and differentiation 7. Upon epidermal growth factor (EGF) binding, activated EGFR triggers phosphorylation and activation of downstream signaling pathways, such as the mitogen-activated protein kinase (MAPK) and phosphatidylinositol 3'-kinase (PI3K)/Akt, which contribute to metastasis, anti-apoptosis, and angiogenesis 7. EGFR overexpression has been found in multiple tumors including breast cancer, lung cancer, and OSCC 8, 9. For example, a high EGFR gene copy number is common in patients with OSCC and oral premalignant lesions 10. *EGFR* amplification is associated with HPV infection, smoking, and poor overall survival in OSCC 11, 12. Yokokawa *et al.* demonstrated that the high co-expression of EGFR and c-Met, another RTK mesenchymal-epithelial transition factor, is a strong prognostic factor in OSCC, with a survival rate below 22% 13. The knockdown of EGFR or the use of an EGFR/c-Met inhibitor induces cell death enhances radio/chemo-sensitivity in OSCC cells 14, 15. For example, the EGFR inhibitor PD153035 inhibits OSCC cell proliferation induced by the carcinogen 7,12-dimethyl benz[*a*]anthracence via downregulation of p-EGFR and p-STAT3* in vivo* 16. Tivantinib, a c-Met inhibitor, causes G2/M cell cycle arrest and apoptosis by downregulating FAK in OSCC cells 17. Iwase *et al.* reported that C225, an anti-EGFR monoclonal antibody, induces Fas-mediated apoptosis and downregulates the expression of p-Akt and c-FLIP 18. Iressa (gefitnib), an FDA-approved EGFR inhibitor for lung cancer therapy, causes G1 cell cycle arrest and suppresses invasion by modulating p27 and MMP-2 in OSCC cells 19. Iressa induces apoptosis and enhances the sensitivity of OSCC cells to cisplatin by suppressing the activation of ERK and Akt 14. Notably, an anti-EGFR antibody-drug conjugate (MRG003) has shown a response rate of 40% in patients with EGFR-positive OSCC in a Phase I clinical trial 20. {*N*-[3-chloro-4-[5-[3-[[[4-[(cyclopropylcarbonyl)amino]3-(trifluoromethyl)phenylamino]carbonyl]-amino]phenyl]-1,2,4-oxadiazol-3-yl]phenyl]-3-pyridine-carboxamide} (COC), a synthetic compound (Fig. 1A), suppresses lung tumor growth by targeting EGFR signaling 21. The dysregulation of EGFR and high co-expression of EGFR and c-Met are strongly associated with the carcinogenesis of oral malignancies and chemoresistance 13, 22. However, there is a lack of information on COC regarding OSCC treatment. Therefore, this study aimed to investigate the anti-proliferative effects and potential molecular target of this compound against OSCC.

## Materials and methods

### Chemicals and antibodies

The chemical synethsis of COC was performed by the co-author Dr. Eman M. E. Dokla as previous report 21. Briefly, a solution containing *N*-[4-[5-(3-aminophenyl)-1,2,4-oxadiazol-3-yl]-3-chlorophenyl]-3-pyridinecarboxamide (0.51 mmol) and phosgene (2.55 mmol) was refluxed in tetrahydrofuran for 4 h under N_2_. Then, the appropriate aniline (0.77 mmol) and *N*,*N*-diisopropylethylamine (1.02 mmol) were added and refluxed overnight. The reaction mixture was concentrated, and the residue was purified by silica gel column chromatography. The identity of COC were verify by nuclear magnetic resonance spectroscopy and high-resolution mass spectrometry. The following antibodies were obtained from Cell Signaling Technology (MA, USA): p38 (#9212), ERK (#9102), EGFR (#4267), MEK (#9122), c-Met (#3148), STAT3 (#9139), JNK (#9252), PARP (#9542), Akt (#9272), mTOR (#2972), p-(Ser473)-Akt (#9271), cleaved caspase 9 (#7237), p-(Ser2448)-mTOR (#2971), p-(Ser217/221)-MEK (#9154), p-(Thr180/Tyr182)-p38 (#9215), p-(Thr202/Tyr204)-ERK (#9101), p-(Ser727)-STAT3 (#9145), cleaved caspase 3 (#9664), and E-cadherin (#3195). β-catenin (GTX101435) and p-(Thr183/Tyr185)-JNK (GTX24821) were purchased from GeneTex, Inc (CA, USA). β-actin (#A5441) was obtained from Sigma-Aldrich (MO, USA), and pro-caspase-8 (#MAB4708) was purchased from Millipore (MA, USA).

### Cell culture

SCC2095 and SCC4 human oral cancer cells were obtained from Prof. Susan R. Mallery and JCRB, respectively. Both cells were cultured in DMEM/F12 medium. Oral fibroblasts were the gifts from Prof. Tzong-Ming Shieh (China Medical University), and were maintained in DMEM medium. All of the cell lines were maintained in a humidified environment at 37^o^C with 5% CO_2_.

### Cell viability

The cell viability of COC was evaluated by the reagent (3-(4,5-dimethylthiazol-2-yl)-2,5-diphenyltetrazolium bromide, MTT). All cell lines (5×10^3^) were seeded into a 96-well plate for overnight. Then, the cells were treated with COC (25, 50, 100, 250, and 500 nM) dissolved in DMSO. After 24 h, MTT reagent was added into the 96-well plate. The plate was incubated for 4 h and the supernatant was discarded 23. Then, the plate filled with DMSO, and the absorbance at 590 nm was determined using a microplate reader (Thermo Scientific Multiskan Go).

### Western blot

Each protein samples (5 μg) were subjected to SDS-PAGE gel for electrophoresis and then electro-transferred to nitrocellulose membranes. The milk containing PBST is used as the blocking solution for these membranes and washed three times by buffer solution. Then, the primary antibodies were incubated with these membranes with shaking for overnight. After washing, the corresponding secondary antibody solutions were incubated with these membranes for 1 h. Then, the membranes were put into an ChemiDOC^TM^ Touch Imaging System (Bio-Rad), where the proteins on the membranes were detected.

### Flow cytometric analysis

Cells (2×10^5^) were treated with COC or staurosporine for 24 h. Then, the Annexin V-FITC/PI apoptosis detection kit was used as the double staining reagents (BD Pharmingen, San Diego), and these cells were determined using a flow cytometer (BD FACSCanto^TM^, BD Biosciences).

### Wound closure assays

SCC4 cells were seeded in 6-well plates for 24 h, the wound was created on the monolayer cells using a micropipette tip, and DMSO or COC was added. The wound was observed and imaged under a phase-contrast microscope.

### The migration assays

The 24-transwell polycarbonate membrane (8 μm) chambers (Corning, USA) were used for the migration* in vitro*. Briefly, SCC4 cells (1.5×10^5^) were seeded into the top chamber with serum-free medium (200 μL) for incubated at 37^o^C for 24 h, DMSO or COC (50, 100 nM) were added. 500 μL of 10% FBS medium were filled in lower chamber. After 24 h, the migrated cells were collected and fixed in methanol (90%) 24. Then, these cells were labeled with crystal violet for 10 min, and were counted in nine fields of vision observed with 100x fields.

### Transfection

Both of the pCMV-Flag (#CV012) and STAT3-CA-Flag (#HG10034-HF) plasmids were purchased from the company (Sino Biological, Pennsylvania, PA, USA). SCC2095 oral cancer cells were transfected with the plasmids (1 μg/well) using Fugene HD transfection reagent (Roche) in a 6-well plate 23. After 24 h, these cells were filled in the appropriate concentrations of COC or DMSO and then the data were detected using Western blot analysis.

### Statistical analysis

The above results were statistically analyzed using Student's *t* tests. A *P* value < 0.05 indicated significant. All of the data were repeated for three tests.

## Results

### COC inhibits OSCC growth

**Figure 2:**
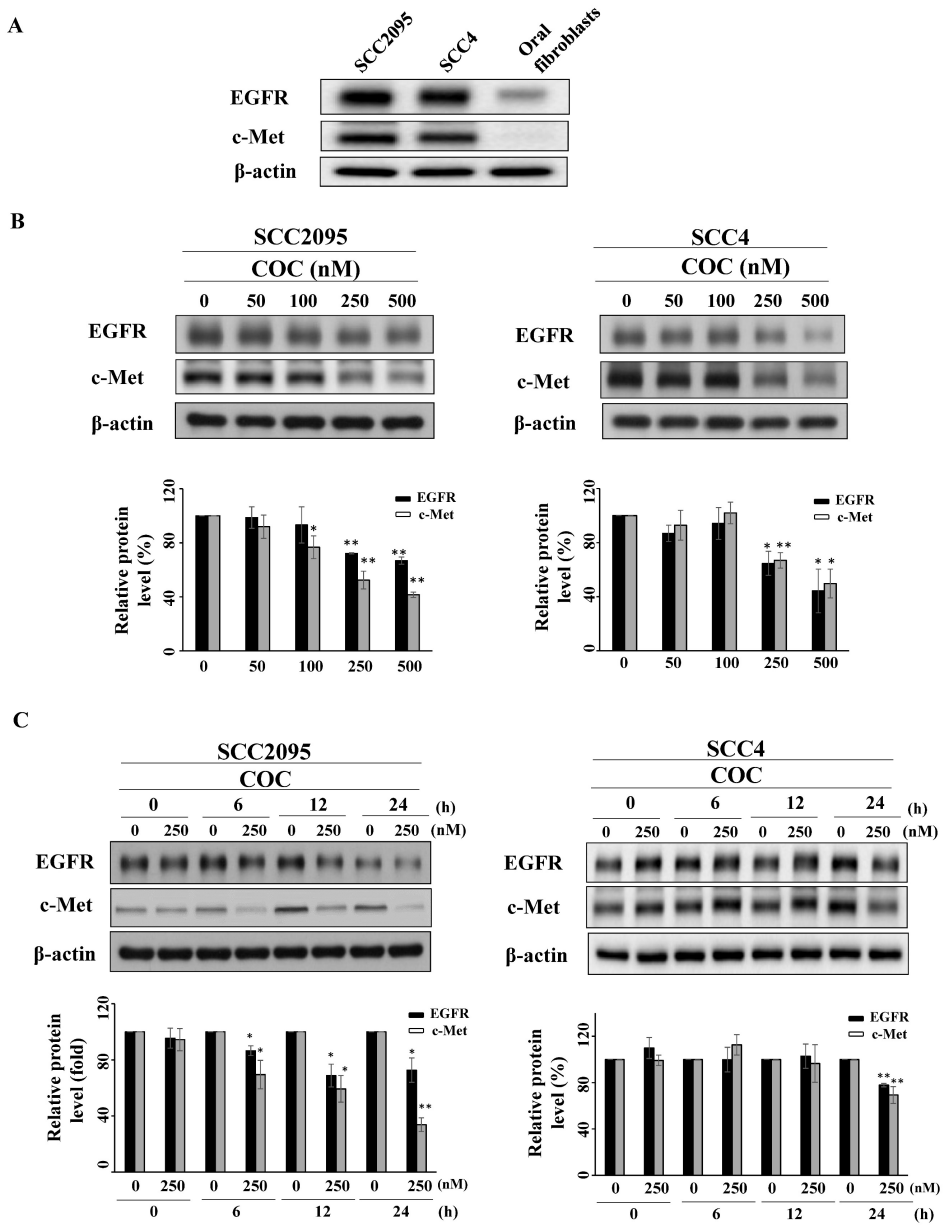
(A) Expression levels of Epidermal growth factor receptor (EGFR) and c-Met in cultured SCC2095, SCC4 oral cancer cells, and oral fibroblasts. Effects of COC on the expression levels of EGFR and c-Met. (B) Concentration- and (C) time-dependent effects of COC on the levels of EGFR and c-Met in OSCC cell lines. *P < 0.05, **P < 0.01.

To examine the inhibitory effects of COC on oral cancer cells, SCC2095 and SCC4 OSCC cells were treated with COC, and cell viability was evaluated. As shown in Fig. 1B, COC decreased the viability of SCC2095 cells in a concentration-dependent manner, with an IC_50_ of 195 nM. COC also inhibited SCC4 cells with an IC_50_ of 204 nM (Table 1). Next, we analyzed the toxicity of COC for 24 h in oral fibroblasts. The results demonstrated that oral fibroblasts were less sensitive to COC, with an IC_50_ of > 500 nM, than SCC2095 and SCC4 cells (Fig. 1C). We also examined the baseline expression of EGFR and c-Met in OSCC cell lines and oral fibroblasts. As shown in Fig. 2A, the two OSCC cell lines expressed relatively high levels of EGFR and c-Met, whereas fibroblasts expressed low levels of EGFR (Table 1). The effects of COC on the expression of EGFR and c-Met in OSCC cells were then evaluated by western blotting. As shown in Fig. 2B, COC decreased the expression levels of EGFR and c-Met in a concentration-dependent manner in both OSCC cell lines. After treatment with COC for 6 h, the expression of EGFR and c-Met in SCC2095 cells decreased (Fig. 2C). Unlike SCC2095 cells, there was no obvious alteration in the expression levels of EGFR and c-Met after treatment with COC in SCC4 cells for 24 h (Fig. 2C).

### COC induces apoptosis in OSCC cells

**Figure 3:**
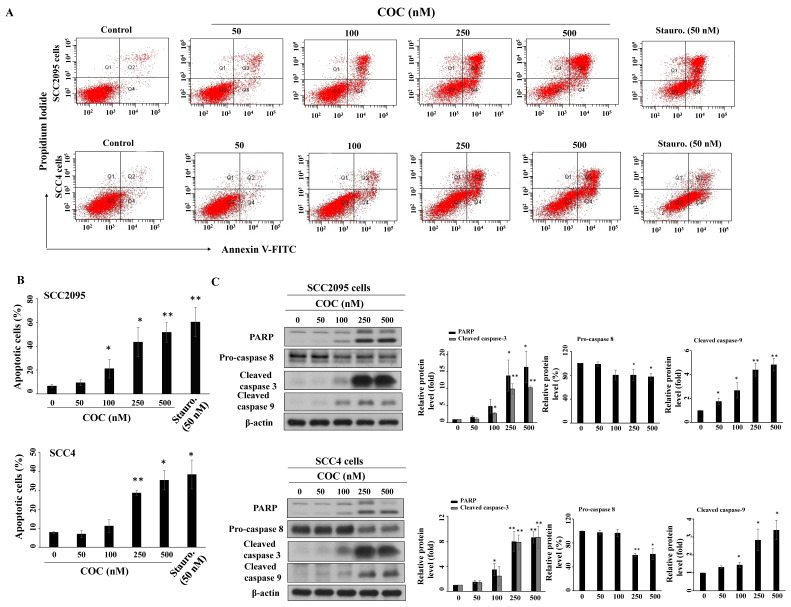
Influence of COC on apoptosis. (A) Apoptotic SCC295 or SCC4 cells after COC treatment for 24 h. Staurosporine (Stauro., 50 nM) was used as a positive control. (B) Statistical analysis of the percentages of apoptotic cells in COC-treated cells. (*n* = 3). (C) Effects of COC on the protein levels of PARP, pro-caspase 8, cleaved caspase 3, and cleaved caspase 9. *P < 0.05, **P < 0.01.

To examine how COC affected cell growth, we investigated the impact of COC on apoptosis. The results show that COC induced apoptosis of SCC2095 and SCC4 cells in a concentration-dependent manner (Fig. 3A). Flow cytometry results revealed that the percentage of apoptotic SCC2095 cells increased from 6.3% to 43.4% after treatment with 250 μM COC (Fig. 3B). A similar trend was also observed in COC-treated SCC4 cells. Western blotting showed that COC promoted the activation of three apoptosis-related proteins, PARP, caspase 9, and caspase 3, through cleavage in a dose-dependent manner (Fig. 3C). Moreover, pro-caspase 8 was downregulated in COC-treated cells, indicating that COC induced caspase-dependent apoptosis (Fig. 3C).

### COC inhibits Akt/mTOR and MAPK signaling molecules

**Figure 4:**
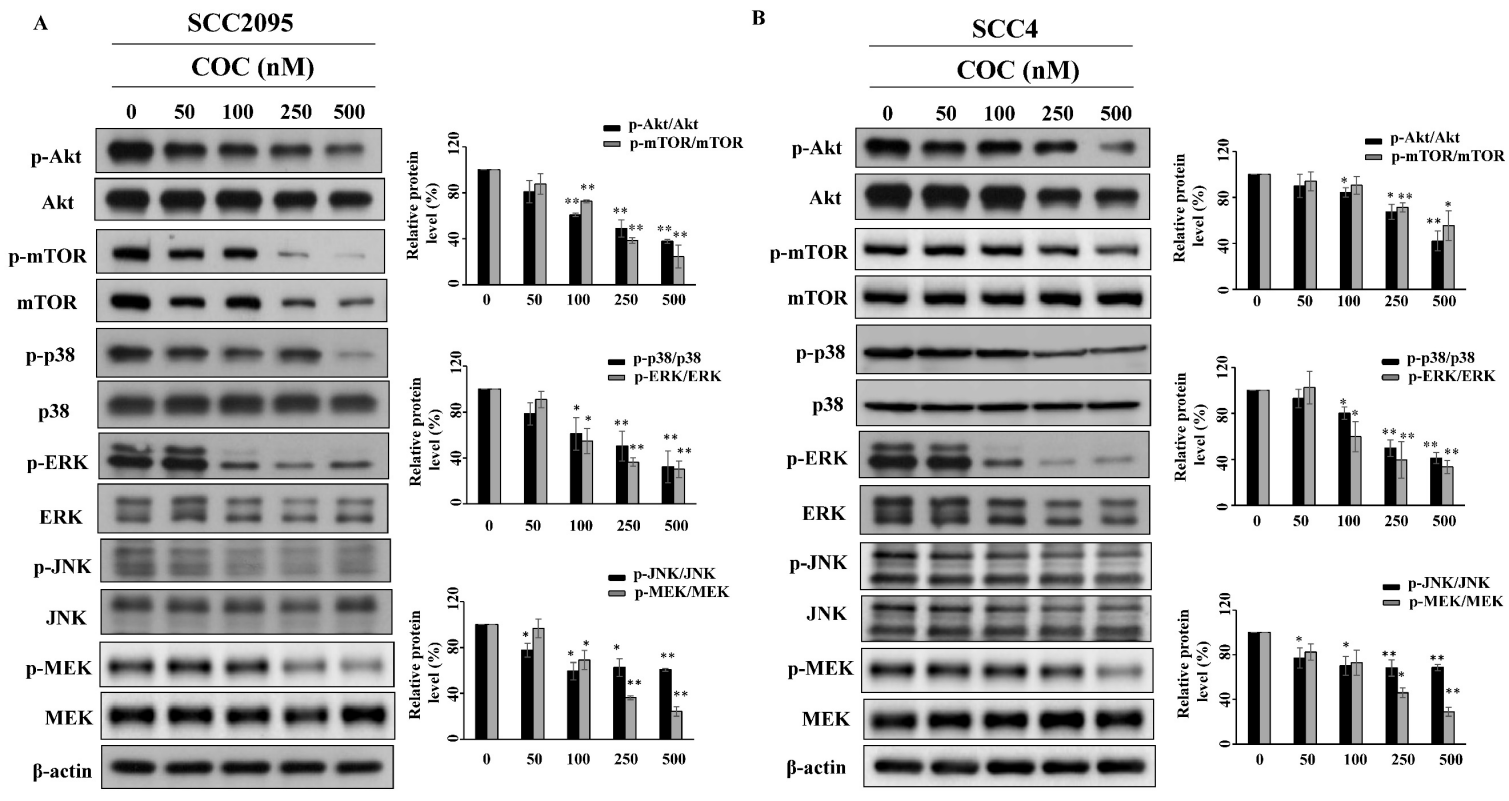
Effects of COC on the protein levels of p-Akt, Akt, p-mTOR, mTOR, p-p38, p38, p-ERK, ERK, p-JNK, JNK, p-MEK, and MEK in (A) SCC2095 and (B) SCC4 cells. Cells were exposed to different concentrations of COC for 24 h. *P < 0.05, **P < 0.01.

Akt and MAPK are part of pathways associated with cell proliferation and tumor progression 25, 26, which led us to evaluate their role in OSCC cells. As shown in Fig. 4A and 4B, COC downregulated the levels of p-Akt (Ser473) and its downstream target p-mTOR (Ser2448) in a concentration-dependent manner. COC also downregulated the MAPK family members p38, JNK, ERK, and MEK (Fig. 4A, 4B).

### STAT3 inhibition by COC leads to cell death

**Figure 5:**
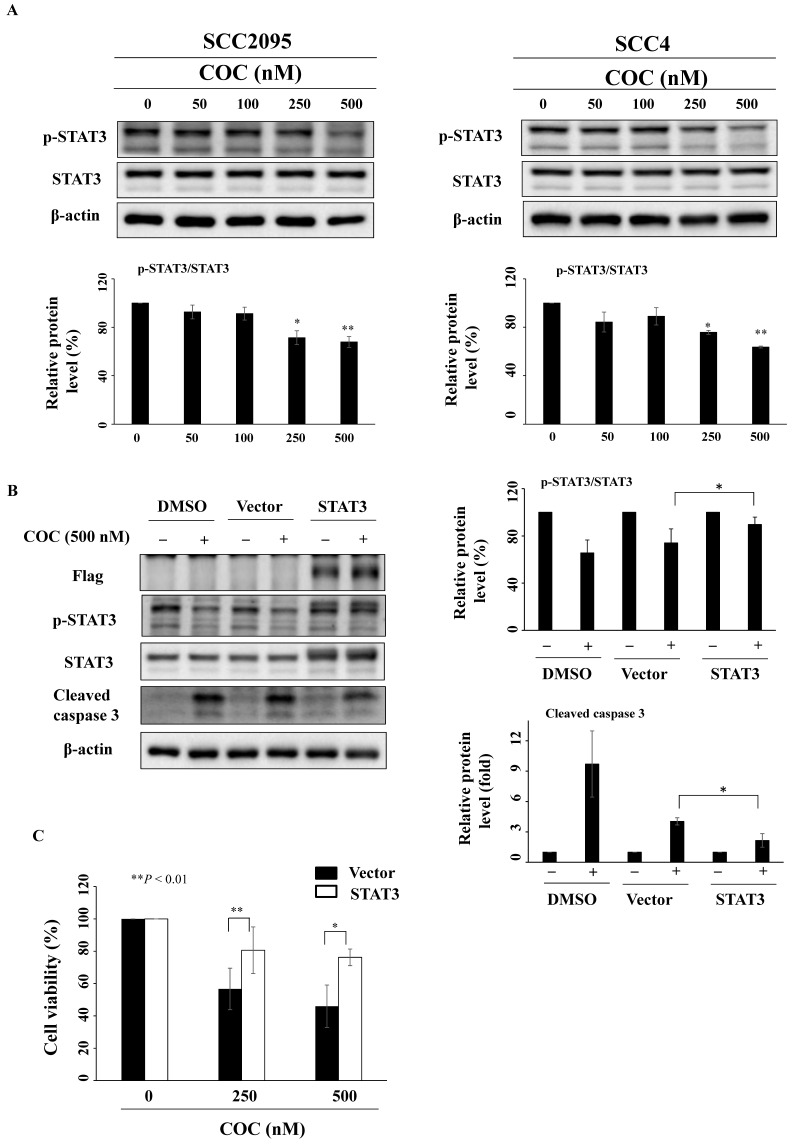
COC downregulates the expression levels of STAT3. (A) Protein levels of p-STAT3 and STAT3 in COC-treated OSCC cells. (B) Effects of STAT3 overexpression in COC-treated cells for 24 h. (C) STAT3-CA partially attenuated COC-mediated cytotoxicity in SCC2095 cells as assessed by MTT assays. *P < 0.05, **P < 0.01.

The oral carcinogen arecoline and the betel nut extract cause oral carcinogenesis through STAT3 activation, which is downstream of the mTOR pathway 27-29. As shown in Fig. 5A, COC downregulated p-STAT3 (Ser727), whereas the total protein expression levels of STAT3 remained constant. To determine whether COC inhibited cell growth by interfering with STAT3, a STAT3 construct was transfected into SCC2095 cells. Western blotting revealed that transfection of the STAT3-CA-Flag plasmid reversed the inhibitory effect of COC on p-STAT3 and cleaved caspase 3 (Fig. 5B). We also observed that the relative cell viability of COC-treated cells was higher than that of wild-type cells (Fig. 5C).

### COC inhibits OSCC cell migration

**Figure 6:**
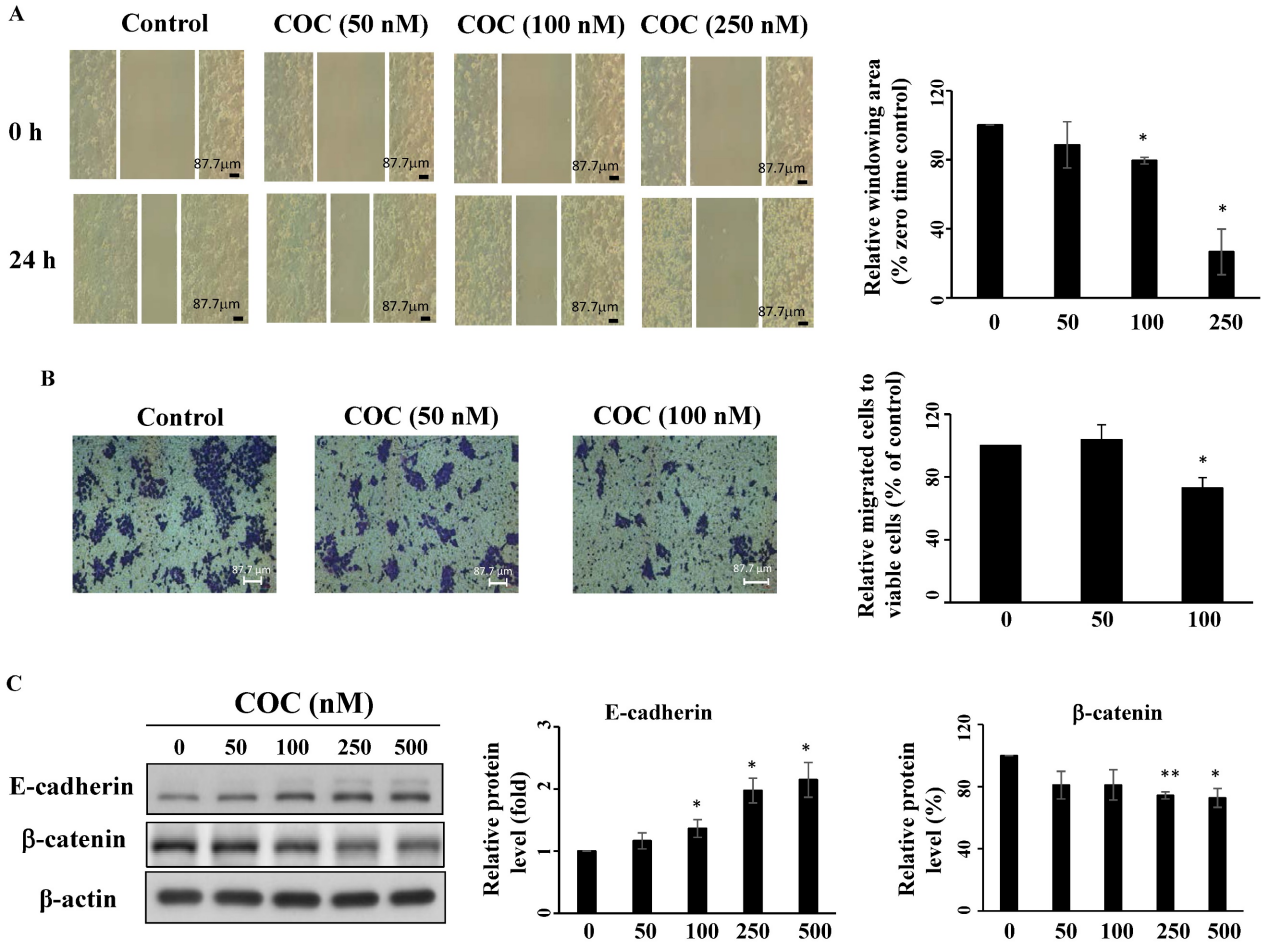
COC inhibits migration of OSCC cells. (A) SCC4 oral cancer cells were treated with COC (50 nM, 100 nM, and 250 nM), and images of wounds were captured by phase contrast microscopy. Magnification ×100. (B) Statistical analysis of the relative window area (%). (*n* = 3). (C) Effects of COC on E-cadherin and β-catenin for 24 h. *P < 0.05, **P < 0.01.

Overexpression of STAT3 promotes tumor progression, therapeutic resistance, and angiogenesis of oral cancer cells 30-32. SCC4 cells show high invasive potential 33; therefore, this cell line was used for migration assays. As shown in Fig. 6A-6B, COC inhibited SCC4 cell migration after treatment for 24 h, as determined by wound closure and Boyden chamber assays. Next, the anti-angiogenesis effects of COC on two epithelial-mesenchymal transition (EMT)-related gene products, including E-cadherin and β-catenin were evaluated by western blotting. The results demonstrated that E-cadherin, the epithelial marker, was upregulated in COC-treated cells (Fig. 6C). For β-catenin, COC decreased the levels of this mesenchymal marker in SCC4 cells (Fig. 6C). We also examined the effects of COC on cell migration in the ectopic overexpression of STAT3. As shown in Fig. S1A, migration increased from 81% to 92 % after COC treatment of STAT3-overexpressing cells. In addition, β-catenin was upregulated in COC-treated cells after transfection of STAT3-CA-Flag plasmid (Fig. S1B). The above results suggested the impact of STAT3 on migration in COC-treated OSCC cells.

## Discussion

**Figure 7:**
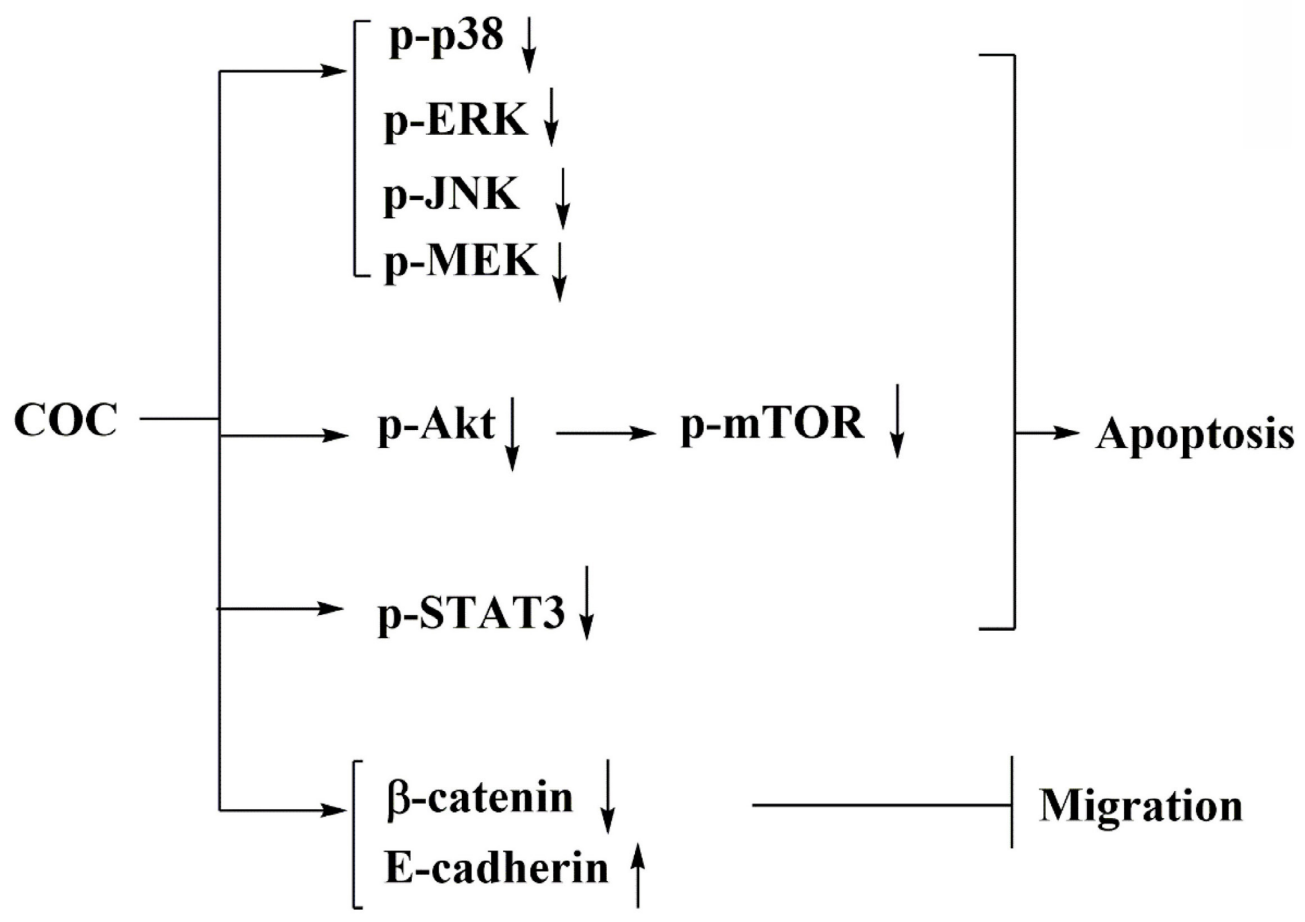
Schematic representation of the pharmacological mechanism of COC-mediated apoptosis in OSCC cells.

Here, we report that COC, a small molecule, induced apoptosis and downregulated Akt/mTOR and MAPK signaling. The IC_50_ for oral fibroblasts was higher than that for OSCC cells, indicating that COC is less toxic to the former cells. In addition, the anti-tumor activity of COC-mediated p-STAT3 inhibition was investigated (Fig. 7).

The majority of chemotherapeutic agents elicit anti-tumor activities via apoptosis 34. During this process, the activation of caspases plays an essential role 35. The death-inducing signaling complex (DISC) and the apoptosome are well-known caspase-activating complexes 35. Poly(ADP-ribose) polymerase (PARP) cleavage by caspase activation is an early recognizable process in apoptosis 36. Linhagen *et al.* reported that Iressa, a chemotherapeutic agent, causes apoptosis in acute myeloblastic leukemia by activating caspase-3 37. In the present study, COC induced caspase-dependent apoptosis and increased PARP cleavage in OSCC cells.

Dysregulation of the PI3K/Akt pathway is involved in the growth of tumors and is associated with the progression of various types of cancers, including oral cancer 38, 39. Iressa inhibits the cell growth of esophageal squamous cell carcinoma by downregulating Akt and inducing apoptosis 40. Our results show that the expression levels of p-Akt and p-mTOR decreased after treatment of OSCC cells with COC. Moreover, the activation of MAPK has been implicated in oral carcinogenesis and is a prognostic biomarker for epithelial malignancies 41, 42. For example, EGF increases the expression of cyclooxygenase-2 (an inflammation-related enzyme) by the activation of ERK and p38 MAPK in OSCC cells 43. Cetuximab induces apoptosis of colorectal cancer cells by inhibiting MEK/ERK signaling 44. p38 MAPK activation contributes to the secretion of pro-inflammatory cytokines on tumor-associated macrophages and OSCC cells 26. In the present study, p-MEK, p-ERK, p-p38, and p-JNK were downregulated after treatment with COC.

The constitutive activation of STAT3 is highly correlated with OSCC progression 45, 46. The accumulation of p-STAT3 has been found in patients with OSCC reporting tobacco consumption but not in the normal mucosa 47. Chen *et al.* reported that all-trans retinoic acid induced apoptosis and decreased PD-L1 expression in OSCC by inhibiting STAT3 48. In our study, COC downregulated p-STAT3 expression in OSCC cells. Furthermore, the ectopic expression of STAT3 partially attenuated COC-mediated cytotoxicity, suggesting that STAT3 is an essential biomarker for COC treatment.

Treatment with STAT3 inhibitors or knockdown of STAT3 inhibits metastasis in various cancers, including OSCC 49-51. Wang *et al.* reported that HJC0152, a STAT3 inhibitor, suppresses the growth and metastasis of OSCC by decreasing p-STAT3 and β-catenin *in vitro* and *in vivo* 52. Oral mesenchymal stem cells promote EMT via the activation of STAT3 and downregulation of E-cadherin, a biomarker of cell adhesion 30, 53. The proinflammatory cytokine IL-6 induces STAT3 phosphorylation, which leads to the interaction between the β-catenin/TCF4 complex and STAT3 signaling 54. Our results show that COC increased the levels of E-cadherin and decreased the levels of β-catenin in SCC4 oral cancer cells. The inhibitory effects of migration and the levels of β-catenin were abrogated after treatment of STAT3-overexpressing OSCC cells with COC, which suggests that STAT3 plays a role in cell migration. Although our results demonstrated that COC inhibited the growth of OSCC via the inhibition of STAT3, a few limitations remain. First, a comparison of COC with chemotherapeutic agents might be valuable for the clinical use of COC. Second, structural modification of COC should be continued. Finally, the *in vivo* efficacy of COC was not evaluated in this study.

## Conclusion

Taken together, our data show that the sensitivity to COC was lower in oral fibroblasts than in OSCC cells. We also demonstrated that COC inhibited STAT3 phosphorylation in oral cancer cells, and these inhibitory effects caused apoptosis and prevented migration. These results improve our understanding of the anti-proliferative mechanisms of COC in OSCC cells.

## Supplementary Material

Supplementary figure.

## Figures and Tables

**Table 1:** Oral cancer cell EGFR and c-Met expression* and sensitivity to COC.

Cell line	Relative expression		COC IC_50_ (nM), mean ± S.D.
	EGFR	c-Met	
SCC2095	High	High	195.3 ± 5.0
SCC4	High	High	203.9 ± 3.0
Fibroblasts	Modest	Low	> 500

*Based on a relative scale of low, modest, and high.

## References

[1] Siegel RL, Giaquinto AN, Jemal A (2024). Cancer statistics, 2024. CA Cancer J Clin.

[2] Filho AM, Warnakulasuriya S (2024). Epidemiology of oral cancer in South and South-East Asia: Incidence and mortality. Oral Dis.

[3] Hashibe M, Brennan P, Chuang SC, Boccia S, Castellsague X, Chen C (2009). Interaction between tobacco and alcohol use and the risk of head and neck cancer: pooled analysis in the International Head and Neck Cancer Epidemiology Consortium. Cancer Epidemiol Biomarkers Prev.

[4] Metri K, Bhargav H, Chowdhury P, Koka PS (2013). Ayurveda for chemo-radiotherapy induced side effects in cancer patients. J Stem Cells.

[5] Contrera KJ, Zafereo ME, Yaniv D, Roberts DB, Gillenwater AM, Hanna EY (2022). Outcomes for recurrent oral cavity squamous cell carcinoma. Oral Oncol.

[6] Makarewicz J, Kaźmierczak-Siedlecka K, Sobocki BK, Dobrucki IT, Kalinowski L, Stachowska E (2024). Anti-cancer management of head and neck cancers and oral microbiome-what can we clinically obtain?. Front Cell Infect Microbiol.

[7] Grant S, Qiao L, Dent P (2002). Roles of ERBB family receptor tyrosine kinases, and downstream signaling pathways, in the control of cell growth and survival. Front Biosci.

[8] Gaye E, Penel N, Lebellec L (2022). Novel treatment approaches for HER2 positive solid tumors (excluding breast cancer). Curr Opin Oncol.

[9] Kang JJ, Ko A, Kil SH, Mallen-St Clair J, Shin DS, Wang MB (2023). EGFR pathway targeting drugs in head and neck cancer in the era of immunotherapy. Biochim Biophys Acta Rev Cancer.

[10] Taoudi Benchekroun M, Saintigny P, Thomas SM, El-Naggar AK, Papadimitrakopoulou V, Ren H (2010). Epidermal growth factor receptor expression and gene copy number in the risk of oral cancer. Cancer Prev Res (Phila).

[11] Chuerduangphui J, Pientong C, Patarapadungkit N, Chotiyano A, Vatanasapt P, Kongyingyoes B (2017). Amplification of EGFR and cyclin D1 genes associated with human papillomavirus infection in oral squamous cell carcinoma. Med Oncol.

[12] Koo K, Mouradov D, Angel CM, Iseli TA, Wiesenfeld D, McCullough MJ (2021). Genomic Signature of Oral Squamous Cell Carcinomas from Non-Smoking Non-Drinking Patients. Cancers (Basel).

[13] Yokokawa M, Morita KI, Oikawa Y, Kayamori K, Sakamoto K, Ikeda T (2020). Co-expression of EGFR and MET has a synergistic effect on the prognosis of patients with oral squamous cell carcinoma. J Oral Pathol Med.

[14] Khalil A, Jameson MJ (2017). The EGFR Inhibitor Gefitinib Enhanced the Response of Human Oral Squamous Cell Carcinoma to Cisplatin In Vitro. Drugs R D.

[15] Zhang M, Han N, Jiang Y, Wang J, Li G, Lv X (2018). EGFR confers radioresistance in human oropharyngeal carcinoma by activating endoplasmic reticulum stress signaling PERK-eIF2α-GRP94 and IRE1α-XBP1-GRP78. Cancer Med.

[16] Ge H, Liu H, Fu Z, Sun Z (2012). Therapeutic and preventive effects of an epidermal growth factor receptor inhibitor on oral squamous cell carcinoma. J Int Med Res.

[17] Xi WH, Yang LY, Cao ZY, Qian Y (2015). Tivantinib (ARQ-197) exhibits anti-tumor activity with down-regulation of FAK in oral squamous cell carcinoma. Biochem Biophys Res Commun.

[18] Iwase M, Takaoka S, Uchida M, Yoshiba S, Kondo G, Watanabe H (2008). Epidermal growth factor receptor inhibitors enhance susceptibility to Fas-mediated apoptosis in oral squamous cell carcinoma cells. Oral Oncol.

[19] Lee EJ, Whang JH, Jeon NK, Kim J (2007). The epidermal growth factor receptor tyrosine kinase inhibitor ZD1839 (Iressa) suppresses proliferation and invasion of human oral squamous carcinoma cells via p53 independent and MMP, uPAR dependent mechanism. Ann N Y Acad Sci.

[20] Qiu MZ, Zhang Y, Guo Y, Guo W, Nian W, Liao W (2022). Evaluation of Safety of Treatment With Anti-Epidermal Growth Factor Receptor Antibody Drug Conjugate MRG003 in Patients With Advanced Solid Tumors: A Phase 1 Nonrandomized Clinical Trial. JAMA Oncol.

[21] Dokla EME, Fang CS, Abouzid KAM, Chen CS (2019). 1,2,4-Oxadiazole derivatives targeting EGFR and c-Met degradation in TKI resistant NSCLC. Eur J Med Chem.

[22] Kanemaru A, Shinriki S, Kai M, Tsurekawa K, Ozeki K, Uchino S (2022). Potential use of EGFR-targeted molecular therapies for tumor suppressor CYLD-negative and poor prognosis oral squamous cell carcinoma with chemoresistance. Cancer Cell Int.

[23] Chu PC, Dokla EME, Hu JL, Weng JR (2022). Induction of apoptosis using ATN as a novel Yes-associated protein inhibitor in human oral squamous cell carcinoma cells. Environ Toxicol.

[24] Omar HA, Arafa el SA, Salama SA, Arab HH, Wu CH, Weng JR (2013). OSU-A9 inhibits angiogenesis in human umbilical vein endothelial cells via disrupting Akt-NF-κB and MAPK signaling pathways. Toxicol Appl Pharmacol.

[25] Hosseini FS, Ahmadi A, Kesharwani P, Hosseini H, Sahebkar A (2024). Regulatory effects of statins on Akt signaling for prevention of cancers. Cell Signal.

[26] Li Z, Liu FY, Kirkwood KL (2020). The p38/MKP-1 signaling axis in oral cancer: Impact of tumor-associated macrophages. Oral Oncol.

[27] Ji WT, Chuang YC, Chen HP, Lee CC, Chen JY, Yang SR (2014). Areca nut extracts exert different effects in oral cancer cells depending on serum concentration: A clue to the various oral alterations in betel quid chewers. Toxicol Rep.

[28] Chuerduangphui J, Ekalaksananan T, Chaiyarit P, Patarapadungkit N, Chotiyano A, Kongyingyoes B (2018). Effects of arecoline on proliferation of oral squamous cell carcinoma cells by dysregulating c-Myc and miR-22, directly targeting oncostatin M. PLoS One.

[29] Ma J, Meng Y, Kwiatkowski DJ, Chen X, Peng H, Sun Q (2010). Mammalian target of rapamycin regulates murine and human cell differentiation through STAT3/p63/Jagged/Notch cascade. J Clin Invest.

[30] Liu C, Zhou J, Zhang S, Fu J, Li Y, Hao Y (2024). Mesenchymal stem cells-derived IL-6 promotes invasion and metastasis of oral squamous cell carcinoma via JAK-STAT3 signalling. Oral Dis.

[31] Wu D, Cheng J, Sun G, Wu S, Li M, Gao Z (2016). p70S6K promotes IL-6-induced epithelial-mesenchymal transition and metastasis of head and neck squamous cell carcinoma. Oncotarget.

[32] Zhang E, Li Z, Xu Z, Duan W, Sun C, Lu L (2015). Frizzled2 mediates the migration and invasion of human oral squamous cell carcinoma cells through the regulation of the signal transducer and activator of transcription-3 signaling pathway. Oncol Rep.

[33] Hall RC, Ayat NR, Qiao PL, Vaidya AM, Ma D, Aminoshariae A (2020). Preclinical Assessment of the Effectiveness of Magnetic Resonance Molecular Imaging of Extradomain-B Fibronectin for Detection and Characterization of Oral Cancer. Mol Imaging Biol.

[34] Russo A, Terrasi M, Agnese V, Santini D, Bazan V (2006). Apoptosis: a relevant tool for anticancer therapy. Ann Oncol.

[35] Park HH (2012). Structural features of caspase-activating complexes. Int J Mol Sci.

[36] Koh DW, Dawson TM, Dawson VL (2005). Mediation of cell death by poly(ADP-ribose) polymerase-1. Pharmacol Res.

[37] Lindhagen E, Eriksson A, Wickström M, Danielsson K, Grundmark B, Henriksson R (2008). Significant cytotoxic activity in vitro of the EGFR tyrosine kinase inhibitor gefitinib in acute myeloblastic leukaemia. Eur J Haematol.

[38] West KA, Castillo SS, Dennis PA (2002). Activation of the PI3K/Akt pathway and chemotherapeutic resistance. Drug Resist Updat.

[39] Osaki M, Oshimura M, Ito H (2004). PI3K-Akt pathway: its functions and alterations in human cancer. Apoptosis.

[40] Teraishi F, Kagawa S, Watanabe T, Tango Y, Kawashima T, Umeoka T (2005). ZD1839 (Gefitinib, 'Iressa'), an epidermal growth factor receptor-tyrosine kinase inhibitor, enhances the anti-cancer effects of TRAIL in human esophageal squamous cell carcinoma. FEBS Lett.

[41] Mishima K, Yamada E, Masui K, Shimokawara T, Takayama K, Sugimura M (1998). Overexpression of the ERK/MAP kinases in oral squamous cell carcinoma. Mod Pathol.

[42] Tashiro K, Oikawa M, Miki Y, Takahashi T, Kumamoto H (2020). Immunohistochemical assessment of growth factor signaling molecules: MAPK, Akt, and STAT3 pathways in oral epithelial precursor lesions and squamous cell carcinoma. Odontology.

[43] Husvik C, Bryne M, Halstensen TS (2009). Epidermal growth factor-induced cyclooxygenase-2 expression in oral squamous cell carcinoma cell lines is mediated through extracellular signal-regulated kinase 1/2 and p38 but is Src and nuclear factor-kappa B independent. Eur J Oral Sci.

[44] Troiani T, Napolitano S, Vitagliano D, Morgillo F, Capasso A, Sforza V (2014). Primary and acquired resistance of colorectal cancer cells to anti-EGFR antibodies converge on MEK/ERK pathway activation and can be overcome by combined MEK/EGFR inhibition. Clin Cancer Res.

[45] Chan KS, Carbajal S, Kiguchi K, Clifford J, Sano S, DiGiovanni J (2004). Epidermal growth factor receptor-mediated activation of Stat3 during multistage skin carcinogenesis. Cancer Res.

[46] Klosek SK, Nakashiro K, Hara S, Li C, Shintani S, Hamakawa H (2004). Constitutive activation of Stat3 correlates with increased expression of the c-Met/HGF receptor in oral squamous cell carcinoma. Oncol Rep.

[47] Macha MA, Matta A, Kaur J, Chauhan SS, Thakar A, Shukla NK (2011). Prognostic significance of nuclear pSTAT3 in oral cancer. Head Neck.

[48] Chen XJ, He MJ, Zhou G (2019). All-trans retinoic acid induces anti-tumor effects via STAT3 signaling inhibition in oral squamous cell carcinoma and oral dysplasia. J Oral Pathol Med.

[49] Liu Z, Li H, Fan S, Lin H, Lian W (2019). STAT3-induced upregulation of long noncoding RNA HNF1A-AS1 promotes the progression of oral squamous cell carcinoma via activating Notch signaling pathway. Cancer Biol Ther.

[50] Jiang M, Li B (2022). STAT3 and Its Targeting Inhibitors in Oral Squamous Cell Carcinoma. Cells.

[51] Xiang M, Kim H, Ho VT, Walker SR, Bar-Natan M, Anahtar M (2016). Gene expression-based discovery of atovaquone as a STAT3 inhibitor and anticancer agent. Blood.

[52] Wang Y, Wang S, Wu Y, Ren Y, Li Z, Yao X (2017). Suppression of the Growth and Invasion of Human Head and Neck Squamous Cell Carcinomas via Regulating STAT3 Signaling and the miR-21/β-catenin Axis with HJC0152. Mol Cancer Ther.

[53] Yadav A, Kumar B, Datta J, Teknos TN, Kumar P (2011). IL-6 promotes head and neck tumor metastasis by inducing epithelial-mesenchymal transition via the JAK-STAT3-SNAIL signaling pathway. Mol Cancer Res.

[54] Yan S, Zhou C, Zhang W, Zhang G, Zhao X, Yang S (2008). beta-Catenin/TCF pathway upregulates STAT3 expression in human esophageal squamous cell carcinoma. Cancer Lett.

