# Supplementary material for: A synthetic molecule targeting STAT3 against human oral squamous cell carcinoma cells
Source: Int J Med Sci. 2025 Feb 10;22(5):1081–91. doi: 10.7150/ijms.105200 (PMC11866527; doi:10.7150/ijms.105200)
Supplement: Supplementary file 1 — Supplementary figure. [file ijmsv22p1081s1.pdf]

Supplementary materials

Fig. S1

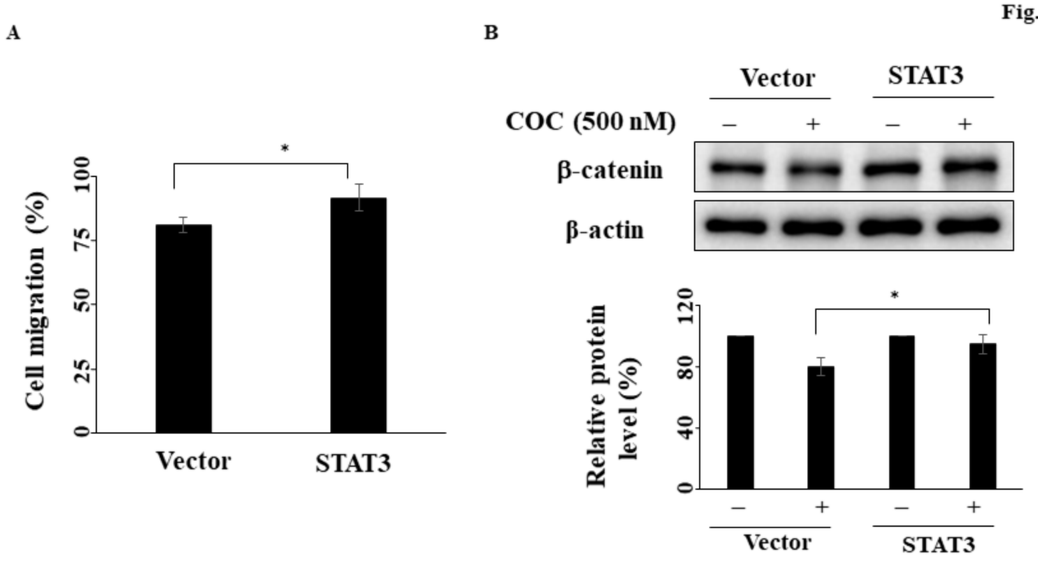

Fig. S1. Effects of STAT3 overexpression on migration in COC-treated cells for 24 h. (A) Left, cells were treated with COC (250 nM) or DMSO after the transfection of the STAT3-CA-Flag plasmids, and images of wounds were captured by phase contrast microscopy. Magnification x 100. Right, the percentage of cell migration. (B) The levels of β-catenin of COC in STAT3-overexpressing SCC4 cells was assessed by Western blotting. *$P < 0.05$.
